# Controlled Breast Cancer Microarrays for the Deconvolution of Cellular Multilayering and Density Effects upon Drug Responses

**DOI:** 10.1371/journal.pone.0040141

**Published:** 2012-06-29

**Authors:** Maria Håkanson, Stefan Kobel, Matthias P. Lutolf, Marcus Textor, Edna Cukierman, Mirren Charnley

**Affiliations:** 1 BioInterface Group, Laboratory for Surface Science and Technology, ETH Zurich, Zurich, Switzerland; 2 Laboratory of Stem Cell Bioengineering, EPF Lausanne, Lausanne, Switzerland; 3 Cancer Biology Program, Fox Chase Cancer Center, Philadelphia, USA; Dresden University of Technology, Germany

## Abstract

**Background:**

Increasing evidence shows that the cancer microenvironment affects both tumorigenesis and the response of cancer to drug treatment. Therefore *in vitro* models that selectively reflect characteristics of the *in vivo* environment are greatly needed. Current methods allow us to screen the effect of extrinsic parameters such as matrix composition and to model the complex and three-dimensional (3D) cancer environment. However, 3D models that reflect characteristics of the *in vivo* environment are typically too complex and do not allow the separation of discrete extrinsic parameters.

**Methodology/Principal Findings:**

In this study we used a poly(ethylene glycol) (PEG) hydrogel-based microwell array to model breast cancer cell behavior in multilayer cell clusters that allows a rigorous control of the environment. The innovative array fabrication enables different matrix proteins to be integrated into the bottom surface of microwells. Thereby, extrinsic parameters including dimensionality, type of matrix coating and the extent of cell-cell adhesion could be independently studied. Our results suggest that cell to matrix interactions and increased cell-cell adhesion, at high cell density, induce independent effects on the response to Taxol in multilayer breast cancer cell clusters. In addition, comparing the levels of apoptosis and proliferation revealed that drug resistance mediated by cell-cell adhesion can be related to altered cell cycle regulation. Conversely, the matrix-dependent response to Taxol did not correlate with proliferation changes suggesting that cell death inhibition may be responsible for this effect.

**Conclusions/Significance:**

The application of the PEG hydrogel platform provided novel insight into the independent role of extrinsic parameters controlling drug response. The presented platform may not only become a useful tool for basic research related to the role of the cancer microenvironment but could also serve as a complementary platform for *in vitro* drug development.

## Introduction

Cancer development and progression is often accompanied by microenvironmental changes that can, in turn, promote (or prevent) neoplasia [1]–[5]. Interestingly, the altered microenvironment has not only been shown to promote cancer progression [6] but also to influence the outcome of treatment. Cell adhesion-mediated drug resistance (CAM-DR) [7] has a transient effect on cell behavior induced for example by extracellular matrix (ECM) signaling. CAM-DR can be primarily attributed to altered cell cycle regulation and/or integrin-mediated survival [8]. Interestingly, tumor-stroma cooperation occurs during cancer progression and often induces CAM-DR. To this end, Sherman-Baust *et al.* demonstrated that over-expression of collagen IV correlates with ovarian cancer grade, while adhesion of tumor cells to collagen IV *in vitro* mediates CAM-DR [9]. Previous research indicates that a similar effect occurred in a β1-integrin dependent manner [10], [11].

In addition to CAM-DR, the dimensionality of the culture environment has been shown to play a central role in the outcome of drug treatment *in vitro* [12]–[14]. It is a general observation that three-dimensional (3D) cell culture, in contrast to two-dimensional (2D) cell culture, better recapitulates the characteristics of the *in vivo* environment [15]–[17]. In cancer, this deviation can partly be explained by the high density of the 3D tumor tissue, which largely affects treatment efficiency by reduced drug penetration over long diffusion distances [18] and can lead to hypoxic conditions. However, it is also hypothesized that the 3D organization per se can alter the cancer cell’s response to apoptotic stimuli [19], even in the absence of oxygen tension differences [20]. For example, the 3D culture may lead to phenotype changes, such as increased levels in cyclin-dependent kinase p27^kip1^ and decreased proliferation [13], which could be explained by increased cell-cell adhesion in 3D [21]. Therefore, we propose that extrinsic parameters important in drug responses and thereby for the explanation of the observed differences between *in vitro* and *in vivo* outcomes include, but are not limited to, dimensionality, extent of cell to cell and cell to matrix interactions and ECM constitution.

The increasing knowledge of the influence of the microenvironment on cancer progression and drug response has initiated an interest in drugs which target the microenvironment [22], [23], [24]. Combinational therapies of traditional chemotherapeutics and targeting of the tumor-stromal interaction to prevent the influence of CAM-DR may not only increase the efficiency of classic therapies but also contribute to the development of a personalized therapy approach. Together with predictive markers, personalized therapy may become the future standard decreasing side effects and increasing efficiency. Specific stromal components are starting to be considered as clinically relevant in various cancers [5], [25]–[27], indicating they could be highly potent biomarkers. In this work, we highlight the need for novel culture models that provide detailed information on the cancer-microenvironment interaction and pave the way to improved pre-clinical models.

A range of different models that mimic the 3D tumor environment have been characterized and regularly used in academia, and lately some of the strategies are being adapted by the pharmaceutical industry [28]. Multi-cellular tumor spheroids have a high complexity and have been shown to recapitulate several characteristics of a non-vascularized tumor [29]. On the other hand, 3D protein matrices are superior at mimicking specific aspects of the cancer cell to ECM interactions, and co-culture systems may be necessary to study processes such as mammary tissue morphogenesis [30]. The growth of cells in Matrigel [31], collagen I [15] or fibronectin-based cell-derived matrices [32] have been irreplaceable for numerous discoveries related to the understanding of matrix-dependent cancer progression [5] and drug response [33]. However, both the spheroids and the 3D protein matrices represent models in which extrinsic parameters, such as three-dimensionality, scaffold rigidity and type of protein coating, cannot be independently controlled. Furthermore, the cell-driven cluster formation in 3D protein matrices makes it difficult to spatially and temporarily control cell positioning. This limits the use of such models in drug development, where microscopy-based read-outs and high-content screening protocols are becoming standard [34].

Therefore, protein-coated microwell arrays can serve as an attractive alternative to standard 3D models, as they permit the culture of cells in 3D adhesive environments with a high control of the culture conditions [35]. This enables the study of the role of different extrinsic parameters, such as dimensionality, matrix coating and the extent of cell-cell contacts independently of each other [36], [37].

Here we investigate the use of a PEG microwell platform for the creation of a multilayer cell cluster microarray with tunable 2D protein coating. By careful selection of extrinsic parameters, a simplified model of tumorigenic breast cancer was achieved, encompassing factors such as cell to matrix and multi-cellular cell to cell interactions. This system enables a high reproducibility in the cancer model fabrication as well as a high control of discrete microenvironmental parameters. This characteristic was used to explore the effect of Taxol against independent extrinsic factors, such as dimensionality, ECM coating and cell density. Our results also clarify the relationship between proliferation and drug response in this context and thereby give some thoughtful information on proliferation rate, cell to cell and cell to matrix interactions as predictive factors.

## Results

### PEG Hydrogel Microwell Arrays as a Platform for High Content Analysis of Multilayer Cell Clusters

It has been established that the PEG hydrogel microwell array is a useful tool to expose cells to controlled microenvironments [38]. We wanted to investigate the suitability of this platform for the formation of confined multilayer clusters to model discrete aspects of the tumor microenvironment. The PEG hydrogels were prepared by micromolding the PEG gel precursor and selectively coating the bottom of the microwells with different ECM proteins to facilitate cell attachment (Fig. S1A). The successful coating of the bottom of the microwells with laminin, collagen I and fibronectin was confirmed by indirect immunofluorescence (see Fig. S1B).

We tested cluster formation in the ECM-coated arrays by culturing the well-characterized human breast cancer cell lines MCF-7 and MDA-MB-231. These cells represent tumorigenic and tumorigenic/invasive breast cancers, respectively. In both cases, cells proliferated and formed multilayer clusters in the microwells within 24 hrs independently of the type of ECM protein coat used (Fig. S2A). However, MDA-MB-231 cells showed a significantly greater reduction in proliferation in comparison to growth on collagen-coated 2D plastic (68±6% in comparison to only 42±7% in MCF-7 cells (see Fig. S2B). Thus, changing the dimensionality of the microenvironment had a greater impact on the proliferation of MDA-MB-231 cells versus MCF-7 cells. Furthermore, the cell density observed in multilayer cell clusters formed of MCF-7 cells was significantly greater in comparison to the density observed in MDA-MB-231 multilayer cell clusters (39±11% greater, p = 0.01) (Fig. S2C).

**Figure 1:**
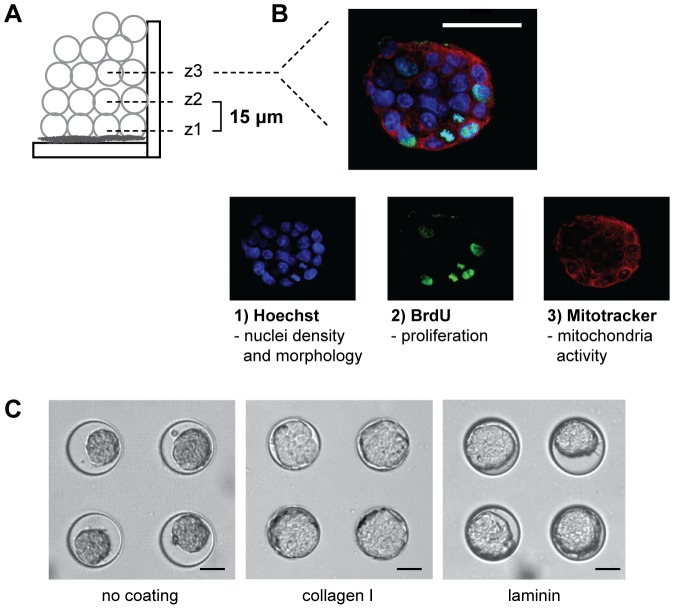
Microscopy-based read-out of the clusters in the microwell array. As the cell clusters in the microwell array are aligned in the same z-plane, confocal imaging can be used to read-out cell behavior. The clusters were imaged at three different topographical planes; z1, z2 and z3 (A). This enables the evaluation of cell behavior at the single cell level and therefore the determination of multiple parameters (B). The lower right image shows nuclear fragmentation and BrdU incorporation levels within a cluster, which were used to assess apoptosis and proliferation, respectively, after treatment with taxol. Scale bar is 50 µm. (C) With this platform, cancer cell clusters formed with similar morhology independent of the protein coating, as shown here for MCF-7 cells on uncoated controls, collagen I and laminin. Note that in the uncoated control wells, multilayer cell cluster spread to a lesser extent in the x-y plane.

Notably, our setup is compatible with conventional inverted confocal microscopy as the thickness of the hydrogel was ≤100 µm and it was molded on a thin support comparable to a cover glass, thickness No.1.5. Thereby, images with sub-cellular resolution from within the clusters (Fig. 1A) could be obtained enabling high content information on the cell state as well as information of the spatial distribution in three dimensions (Fig. 1B).

### Cell to Matrix Interaction Plays a Key Role in the Multilayer Cell Clusters’ Response to Drug Treatment

The importance of cell to matrix interactions, matrix composition and the 3D organization of cells in the regulation of drug response has been highlighted in the literature [11], [33]. For this reason we hypothesized that the controlled environment of the microwells could be a valuable tool to retrieve more information on the role of matrix interaction in multilayer cell clusters. In particular, the platform enabled the study of matrix effects independently of other factors. Indeed, the initial results showed that MCF-7 cell clusters representing early breast cancer showed no matrix-dependent morphology differences between laminin and collagen I (Fig. 1C). Independently of the protein coating in the 90 µm wide microwells, clusters were formed with a width ranging between 80–90 µm. The height of the clusters was determined to 56±3 µm at 48 hr after seeding (Fig. S3). In controls without matrix coating, the multilayer cell cluster spread less in the x-y plane and were substantially smaller in size (diameter ranging between 55–65 µm). This suggests that spreading of multilayer cell cluster was due to the protein coating, and, in control samples without the protein coating, there was no effective interaction with the PEG hydrogel (Fig. 1C).

**Figure 2:**
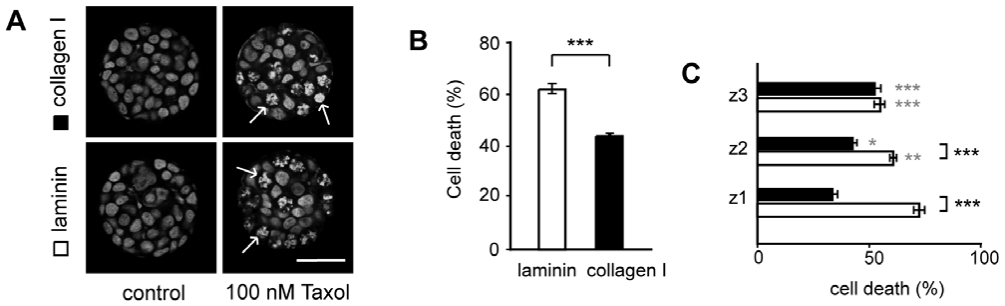
Matrix-dependent drug response in multilayered cultured cells. Cell death after Taxol treatment was determined by analysis of nuclear morphology in confocal images. The example images in (A) are representative images of cells in the z2-section stained with propidium iodide after fixing in methanol. A significantly higher number of fragmented nuclei were observed following Taxol treatment in cell clusters cultured in both collagen I and laminin-coated microwells. The response to 24 h treatment with 100 nM Taxol in MCF-7 clusters after 24 h cluster formation was significantly higher in cells cultured in laminin-coated wells compared to cells cultured in microwells coated with collagen I (B). To further understand the effect of the matrix on drug response, we assessed drug response at different locations within the clusters. The difference between laminin and collagen I at 24 h was predominant at the two lower z-positions, z1 and z2 (C). Further away from the matrix coating, at the z3 location, the cell death was not matrix coating-dependent. Scale bar is 50 µm. (*, ** and *** = p<0.05, p<0.01 and p<0.001 respectively, ns  =  not significant. The grey stars represent significant differences between z1 and the other image planes for laminin and collagen I respectively. Black stars represent comparisons between laminin and collagen I).

Interestingly, matrix effects, as previously observed in unconfined monolayer cultures, could be reproduced in the multilayer clusters. After 24 h exposure to 100 nM Taxol, cell death in the clusters was determined by analysis of nuclear morphology (Fig. 2A). In collagen I-coated microwells, cell death was 19±2% lower (p<0.001) than in wells with laminin coating (Fig. 2B). These results suggest that the specific interaction with the mesenchymal protein may impart a Taxol protective or Taxol resistance effect.

### The Effect of the ECM Depends on the Position of the Cell within the Cluster

To further understand the role of the specific matrix proteins on Taxol response, we quantified the level of cell death as a function of the specific cell position within the clusters (i.e. determining the amount of cell death at the individual image planes z1, z2 and z3, corresponding to bottom, middle and top sections, respectively).

As expected, the largest difference between collagen I and laminin occurred at the bottom layers of the clusters where direct cell to matrix interactions were predominant (Fig. 2C). On laminin, the drug response increased as the matrix interactions became more prevalent (18±4% increase in cell death from z3 to z1, p<0.001) while collagen I showed the reverse trend (19±2% decrease in cell death from z3 to z1, p<0.001). The drug response at plane z1 (bottom) was 39±4% higher on laminin compared to on Collagen I (p<0.001). A smaller difference, but following the same trend, was observed in image plane z2, in which the cell death was 16±3% higher on laminin (p<0.001). Interestingly, at a distance of 35 µm away from the matrix interface in the z3 slice, there was no significant difference in the response to Taxol on laminin vs. collagen I.

### Inhibition of β1-integrin Increases Taxol Response in the Multilayer Cell Clusters

Previous work has highlighted the importance of integrin β1, i.e. the two major collagen receptors α1β1 and α1β2 [39], for proliferation, survival and invasive signaling in breast cancer cells [32], [40], [41]. Thus, we decided to explore the role of β1-integrin in the observed Taxol responses. This was achieved by treating the cell clusters with the well-characterized monoclonal antibody 13 (mAb13) that binds to integrin-β1 and favors its inactive conformation [42].

**Figure 3:**
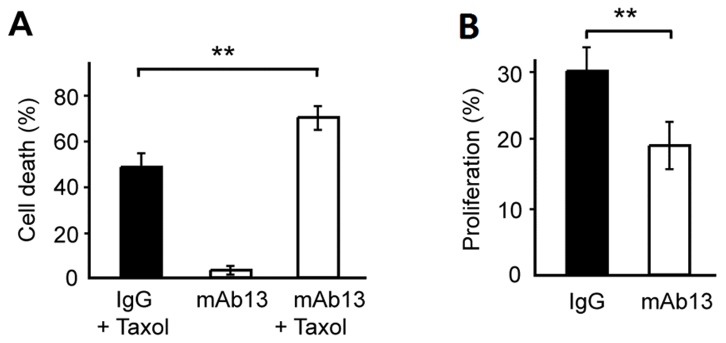
The effect of blocking β1-integrin on drug response and proliferation. MCF-7 multilayer clusters cultured in collagen I-coated microwells were treated with the integrin-blocking antibody mAb13 and/or Taxol. The percentage of cell death (A) and proliferation (B) are depicted. Note how mAb13 treatment significantly increased cell death, while integrin-blocking also affected the proliferation when compared with IgG controls. (** = p<0.01).

When β1-integrin binding was inhibited in combination with Taxol treatment, the average cell death was increased by 21±5% (p<0.01) in comparison to controls treated with Taxol and an unspecific IgG antibody (Fig. 3A). Hence, this data suggests that the interaction with collagen I induced a protective effect on the cancer cells reducing their response to Taxol even after 48 hrs culture. Furthermore, it indicates that β1-integrin plays a major role in this adhesion-mediated effect. Treatment with mAb13 alone did not lead to a significant increase in apoptosis; cell death was consistently below 3% during mAb13 treatment (Fig. 3A). This shows that the combinatorial effect of Taxol treatment and β1-integrin-blocking was not cumulative but rather synergistic. Intriguingly, the effect of β1-integrin blocking varied according to the position of the cell within the multilayer cluster (Fig. S4).

The effects of β1-integrin blocking on drug response were in extension correlated to proliferation levels as proliferation rate closely relates to Taxol response. In fact, mAb13 treatment per se significantly reduced the average proliferation by 10±3% (p<0.01) (Fig. 3B).

### Dimensionality-related Differences in Drug Response are Markedly Reduced when Cell Density in Mono- and Multilayer Clusters is Comparable

**Figure 4:**
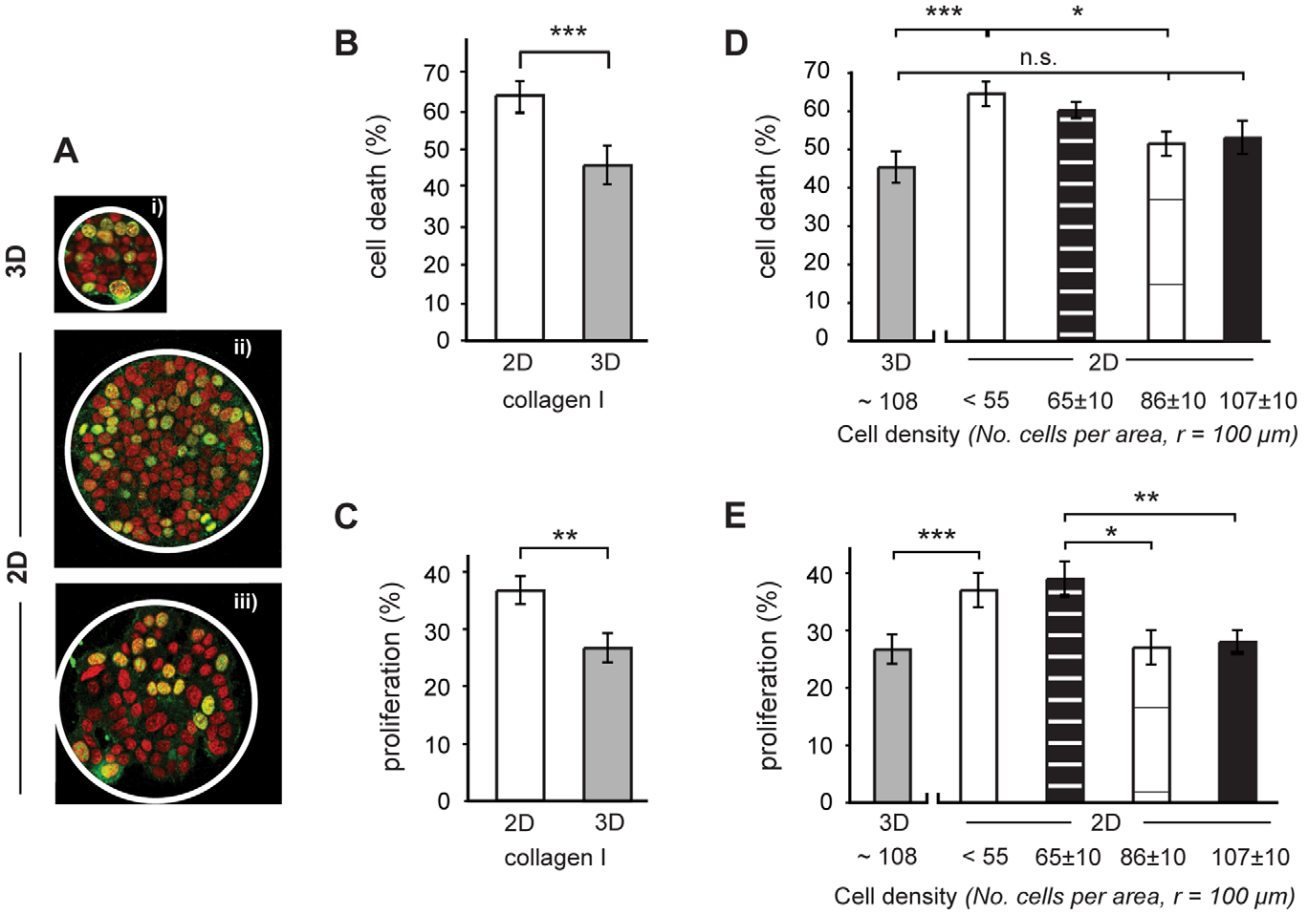
The role of cell density on drug response in multilayered cell culture. The effect of cell density was determined by comparing cells at high cell density in multilayer cell clusters to cells cultured at different densities in monolayer clusters. The images in (A) show cells at the different experimental conditions. To determine proliferation levels the cells were stained with a primary antibody for BrdU incorporation (green) and their nuclei were counterstained with PI (red). Here, (i) shows cells in 90 µm wide 3D clusters at high density while (ii) and (iii) are cells cultured on the 200 µm wide (A = 3×10^4^ µm) 2D patterns at high and low density, respectively. Initially, it was confirmed that the response to treatment with 100 nM Taxol was significantly lower in the multilayer clusters compared to cells grown on flat hydrogel substrates (B). Similarly proliferation levels, measured by BrdU incorporation, was lower in the multilayers (C). In the next step, we tested the role of cell density on drug response by culturing cells as confined monolayers on collagen I patterns. It was found that drug response was significantly reduced with increasing cell density (D) in the monolayers. When the cell density was matched in the mono- and multilayers, there was only a small difference in drug response which was not significant. Using the same experimental setup, we found that proliferation in monolayers decreased with increasing cell density (E). A significant decrease in proliferation compared to a sub-confluent situation was observed at a cell density of 86 cells per pattern. At matching densities in the mono- and multilayer clusters, there was no significant difference in proliferation. (*, ** and *** = p<0.05, p<0.01 and p<0.001 respectively, ns  =  not significant).

It has been repeatedly shown that 3D culture reduces the response to drugs [14]. While many microenvironmental parameters may differ substantially in 3D vs. 2D, we decided to use our controlled model system to elucidate the role of a few defined parameters. By comparing cells cultured as multilayer cell clusters in 90 µm wide collagen coated microwells to cells cultured as monolayer clusters on 200 µm wide collagen patterns, we were able to assess the roles of cell density and dimensionality independently of other parameters (Fig. 4A).

In line with previous evidence, we observed that the drug response was significantly lower in the multilayer cell clusters in the microwell array compared to cells cultured as unconfined monolayers on a hydrogel without patterns (45±4% and 63±2% cell death respectively, p<0.001) (Fig. 4B). Furthermore, it was found that the observed reduced drug response in the multilayer clusters correlated with a reduction in cell proliferation (Fig. 4C). Control cells proliferated at a 10±4% lower rate in multilayers compared to in monolayers (p<0.05). To elucidate the effect of cell density in these differences, we exploited the natural variation in cell density on circular collagen-I patterns (Ø = 200 µm) on the microstructured PEG hydrogel. The confined monolayer clusters allowed us to control the cell density, i.e. the surface area of contact between neighboring cells. For analysis of the results, we binned the cell numbers into four categories ranging from low, sub-confluent density with <55 cells per pattern to high cell density with approximately 100 cells per pattern. Importantly, the cell number at the highest density was comparable to the cell density in a multilayer cluster after 48 h culture. Using this experimental setup, it was found that drug response in the confined monolayers significantly decreased with increasing cell density. At the lowest cell density (<55 cells per pattern), the cell death was 63±3%, which is 11±4% higher than at a cell density of 76–96 cells per pattern (p<0.05) (Fig. 4D). At the highest monolayer cell density, with 97–107 cells per pattern, i.e. similar to the cell density in the multilayer clusters, the cell death was slightly, but not significantly, greater than in the multilayers.

To determine how the observed differences were related to cell proliferation, we repeated the experiment in the absence of drug treatment and determined the density-dependence in proliferation. An effect of cell density on proliferation was first observed at a density of about 86 cells per pattern (Fig. 4E), where proliferation decreased by 12±4% compared to the lowest cell density (p<0.05). As could be expected, the proliferation at the highest cell density with 100 cells per monolayer, which matches the cell density in the multilayer clusters, was not significantly different to the proliferation levels observed in the multilayers.

### Down-regulation of E-cadherin Increases Proliferation at High Cell Densities

The effect of cell density on cell behavior could be explained by several factors such as increased cell-cell contacts and morphology changes of the cell and its nucleus. The results of previous studies have suggested a correlation between increased E-cadherin, growth suppression and reduced drug response in 3D cultured cancer cells [13], [21]. To determine the role of E-cadherin in the cell-density dependent proliferation, we decided to knockdown its expression.

**Figure 5:**
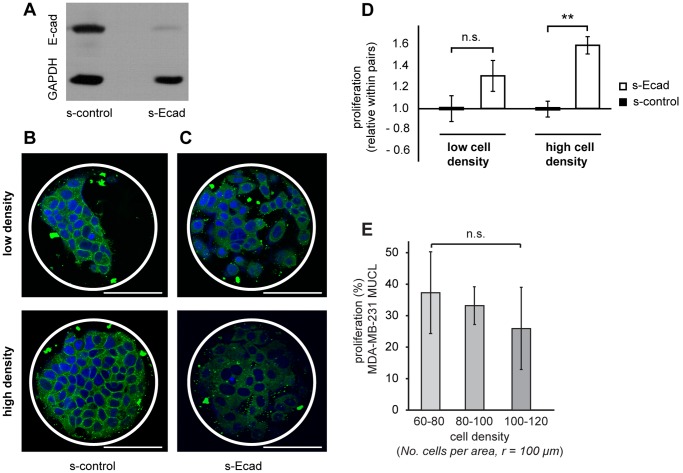
E-cadherin affected cell proliferation but not morphology in MCF-7 cells. E-cadherin knockdown in the MCF-7 cells was confirmed by western blot (A). GAPDH levels were used to account for differences in total cell numbers between samples. The confocal images show cells growing on collagen patterns fixed 48 h after knockdown and stained for E-cadherin (green) and cell nuclei (blue). After knockdown of E-cadherin levels with si-RNA (C), the E-cadherin distribution in the cells was clearly different to cells only treated with s-control (B). Interestingly, when E-cadherin was depleted from the MCF-7 cells, increased proliferation in the monolayer cell clusters was observed (D). The extent of the effect of the knockdown was shown to be dependent on the cell density. At low densities, the relative proliferation increase of 1.3 fold compared to si-RNA control was not significant. Conversely, at high cell densities, the proliferation increased 1.6 fold compared to si-RNA control and was 30% higher than the proliferation levels at low density. Hence, this data indicates that E-cadherin is responsible for the reduced proliferation observed at high cell densities. To further confirm this finding, we investigated the behavior of the MDA-MB-231 cells which do not express E-cadherin. Interestingly, we observed no difference in proliferation in MDA-MB-231 multilayers with different densities (E). (** = p<0.01, ns  =  not significant).

The down-regulation of E-cadherin levels in the MCF-7 cells by E-cadherin siRNA reached an efficiency of 80%, as confirmed by western blot (Fig. 5A). It was found that this down-regulation caused only a modest effect on the cell morphology of cells cultured as monolayer clusters on collagen I patterns (Fig. 5B and C). Following siRNA transfection, cells still grew as colonies with the individual cells in close contact with one another. However, staining E-cadherin by indirect immunofluorescent staining showed clear differences after siRNA treatment. In cells treated with scramble siRNA (s-control), E-cadherin was clearly present at high concentrations at cell-cell contacts (Fig. 5B). Conversely, after exposure to E-cadherin siRNA (s-Ecad), only a homogenous intracellular background staining was observed (Fig. 5C), probably representative of the 80% knockdown efficiency.

Interestingly, the down-regulation of E-cadherin levels at high cell densities caused a significant increase in proliferation (1.6 fold increase) compared to the s-control (Fig. 5D). The relative proliferation increase after knockdown at high vs. low density differed by approximately 30%, which corresponds directly to the relative difference in proliferation between low and high density observed in un-transfected cells (Fig. 4E). The tendency for increased proliferation after knockdown at low cell densities suggests that E-cadherin plays a role in proliferation at these densities. Nonetheless, the results also suggest an increased importance in the effect imparted by E-cadherin expression levels at high cell densities.

To further explore the correlation between proliferation and cell density, we examined the effect of changes in cell density in the E-cadherin negative cell line, MDA-MB-231. Interestingly, although the cell density varied substantially in MDA-MB-231 multilayer clusters, no difference in proliferation was observed (Fig. 5E). Thus, in the absence of E-cadherin, changes in cell density did not seem to impact on cell proliferation supporting the role of E-cadherin in the density dependent effects observed in MCF-7 cells.

### Cell Density Variations Could Not Explain the Matrix-dependent Drug Responses

**Figure 6:**
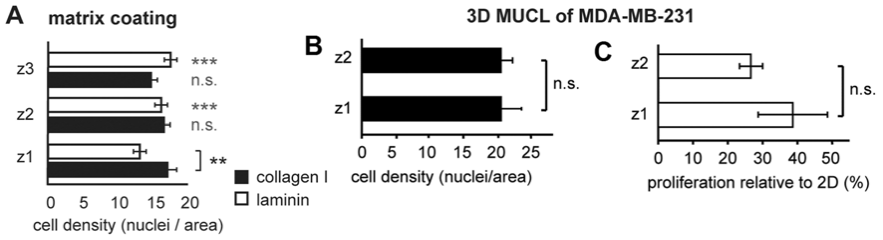
The role of cell density in the matrix-dependent drug response. Cell density was predominately unaffected by either matrix coating or the cell’s position within the multilayer clusters (A). The only observed difference was a slightly lower cell density in z1 for clusters growing on laminin. Hence, any effect of matrix coating could be expected to be independent of cell density. In MDA-MB-231 clusters, there was little variation in the cell density regardless of the z position in the (B). However, a trend towards higher proliferation at the interface with the matrix was observed but not statistically confirmed (C). (MUCL  =  multilayer cell cluster, ** and *** = p<0.01 and p<0.001 respectively, ns  =  not significant. Grey symbols in A represents the comparison to z1).

In the observations depicted above, we confirmed a cell density dependence in drug response. Therefore, we decided to examine the variation in cell density in the data obtained for the investigation of matrix effects. Interestingly, no differences in cell density were observed at any location within the clusters cultured on collagen I (Fig. 6A). This indicates that the observed differences in drug response at the different z-positions (Fig. 2C) were purely matrix-dependent. In comparison, in the laminin-coated microwells, there was a small difference in cell density between the bottom image plane (z1) and the other locations. The cell density was 13±1 cells per area at z1, which is 4.5±1 and 3±1 cells lower than at position z3 and z2 respectively (p<0.001) (Fig. 6A). However, this difference did not correlate with the pattern for the drug response distribution in the clusters, where the highest values were obtained in z1 (Fig. 2C). In the clusters of the invasive MDA-MB-231 cells, there were no significant differences in the cell density regardless of the cells’ position within the microwell (Fig. 6B). However, there was a slight, but insignificant, increase in proliferation at z1 in contact with collagen I (Fig 6C).

## Discussion

It is well accepted that the microenvironment plays an important role in determining the responsiveness of cancer cells to drug treatment [43], [44]. Certain aspects of the microenvironment are key to this role including the dimensionality, adhesive proteins and cell morphology. In numerous 3D cell culture models, it has been observed that cells present in a 3D configuration show lower response to chemotherapeutics in comparison to cells cultured on flat (2D) substrates [12]–[14]. This effect could be linked to reduced cell growth [13], phenotype changes such as increased malignant potential [14] and the limited diffusion of drugs and nutrients [45]. The relationship between E-cadherin, proliferation and cancer drug response has previously been explored through the study of cells in spheroids [21]. On the other hand, the effect of different matrix proteins on drug response has mainly been investigated using 2D protein-coated substrates [11].

A drawback of the majority of current cancer models is their inability to independently control the extrinsic parameters of the microenvironment, either because the parameters are linked or the model system is too complex. An illustrative example of this is the matrix-specific morphology changes observed for breast cancer cells [46]. Therefore, by studying cells on a conventional 2D matrix array, it may be difficult to determine the effect of the signaling from the matrix independently of other factors. To this end, the PEG microwell array allows the production of breast cancer models in which culture parameters can be tightly controlled. Thereby, the extrinsic parameters could be individually manipulated to determine their specific effects upon cell behavior.

### The Role of Cell to Matrix Interactions in Drug Responses within Multilayer Cell Clusters

Initially, we confirmed the formation of multilayer clusters of both MCF-7 and MDA-MB-231 cells within the microwells. MCF-7 cells formed clusters with a narrow size distribution, independent of the protein coating. This is in contrast to previous studies in more traditional 3D cell culture systems, in which clear morphological changes with different matrix proteins were observed [46]–[48]. This system therefore allowed exploration of matrix interaction independently of morphological changes, which is not possible in alternative model systems.

Using a confocal imaging-based readout, it was revealed that cell response to Taxol was clearly dependent on the localization (i.e., z-position) of the cells in question within a given cluster. Cells in contact with collagen I were significantly less responsive to treatment compared to cells present in areas where cell-cell contacts were dominant. Since the cell density was consistent throughout the entire multilayer clusters, we concluded that the observed effect must be purely matrix dependent. An opposite drug response pattern was observed in laminin-coated wells, however the cell density in these clusters did vary, and consequently it was non-trivial to deconvolve the differential effects of cell to matrix and cell to cell interactions. From these results, it can be concluded that the collagen I interaction either directly or indirectly made the cells less responsive to, or protected them from, Taxol treatment. This result is in agreement with observations indicating that breast cancer cells may show reduced drug response after adhesion to collagen [11], [49]. Interestingly, the collagen-specific reduction in drug response, in comparison to laminin, supports the idea that cancer progression promotes higher drug resistance. Collagen I represents a later stage of tumorigenesis where the basement membrane that traditionally separates epithelium from mesenchyme has been damaged or degraded. The reduction in drug response with the z-position in the laminin clusters could be due to other factors such as enhanced cell to cell interactions in the middle of the cluster and lack of specific integrin engagements in the absence of collagen I. On the contrary, MDA-MB-231 cells cultured as multilayer clusters in collagen-coated microwells showed a tendency for higher proliferation at the interface with the protein in a density-independent manner. This could be explained by the proliferation-promoting effect of collagen I [48]. Hence, the strong effect of dimensionality on proliferation in these cells could be interpreted as a lack of matrix adhesion in the multilayers in comparison to monolayer cultures.

The contribution of β1-integrins on the collagen I-dependent effects was confirmed, as blocking this interaction led to a major increase in drug response. This observation correlates with work by Aoudjit, et al., who showed that collagen I-dependent drug response could be related to the collagen I-specific integrin heterodimer α2β1 [11], as well as with our own work, which highlighted the contribution of β1-integrin to cancerous behavior [32], [33], [50]. Interestingly, blocking the β1-integrin also induced reduced cell proliferation indicating an inverse relationship between proliferation and drug response. This last point may be considered important in the study of drug efficacies in cancer, especially in cancer instigating cells, e.g., cancer stem cells that are known for their self renewal capabilities, their strong dependency on a physical ECM containing niche, as well as for their low proliferative rates [51].

### The Role of Cell Density in the Drug Response in Multilayer Cell Clusters

In a central experiment, the new platform was used to study the importance of cell density in dimensionality-dependent drug response. Initially, it was shown that cells in the multilayer clusters were less susceptible to Taxol and demonstrated a reduced proliferation compared to cells cultured as monolayers. Using collagen-patterns with cells at increasing densities, it was found that drug response was clearly density dependent. Furthermore, this data indicates that the increased cell density in the multilayers could largely explain the dimensionality effect typically observed in 3D cultures.

Interestingly, the reduced proliferation rate observed at increased cell density in the monolayers closely approached the low values seen in multilayer clusters. Therefore, it can be concluded that the main reason for a ‘three-dimensionality’ effect in these models of reduced complexity was the increased cell to cell interactions together with a reduced proliferation seen at high cell densities (i.e., both in multilayer cell cluster and highly dense 2D monolayer cell cluster). The knockdown of E-cadherin expression confirmed that the reduced proliferation at high cell densities was tightly linked to the levels of cell to cell interactions. The key role of E-cadherin in this effect is further supported by the absence of cell density effects on proliferation in multilayer clusters formed by the E-cadherin negative cell line MDA-MB-231. It has been previously demonstrated that E-cadherin and growth factor levels are the main determinants of growth, above cell morphology and size, using the controlled culture of normal epithelial cells [52]. The effect of E-cadherin in some cancers has been shown by transfection of E-cadherin and applying E-cadherin neutralizing antibodies to disrupt cell adhesion [21]. However, these methods also affected the global cell morphology. To our best knowledge, this is the first study which shows that in a controlled cancer model E-cadherin-mediated cell-cell adhesion alone is a major factor contributing to the reduced Taxol susceptibility in 3D.

### The Advantage of Controlled Model Systems

The application of the PEG microarray platform for the exploration of the exact role of different extrinsic parameters on drug response demonstrated the advantage of controlled model systems. Clusters within microwells formed and showed no apparent morphological differences due to the types of matrix proteins being used. Therefore, this system enabled the study of matrix-dependent cell behavior independently of parameters such as cell morphology, contact area with the ECM, etc. Also, retaining the spatial location of the cell within the cluster allowed the inter-relationship between cell to matrix vs. cell to cell interactions to be explored within a simplified 3D environment. Finally, the ability to control cell densities enabled us to reveal the role that cell density plays in “three-dimensionality”-dependent effects, as well as the contribution of E-cadherin. The use of this model system enabled us to confirm previously published studies, which demonstrated that proliferation and drug response were reduced in the presence of collagen I or E-cadherin interactions [11], [21], [49], thus validating the use of this platform. Furthermore, the exploitation of the model system permitted us to gain novel insights into the differential effects of cadherin and integrin based adhesions. Therefore, we propose that culturing cells with the possibility of imparting a high level of control of individual extrinsic parameters will facilitate the discovery of key signaling mechanisms responsible for the regulation of the distinctive aspects of the microenvironmental milieu. Furthermore, important inter-relationships between extrinsic parameters may be determined. In future work it would be interesting to use this platform to explore in greater detail the adhesions formed within the multilayer cell clusters and furthermore determine the inter-relationships between integrin and cadherin based signaling and its impact on specific drug responses. Therefore, it is possible that this type of model will complement additional models, such as cancer spheroids or more complex models including organotypic 3D culture systems (amongst others) and animal models, in basic and pre-clinical cancer research.

### Proliferation as a Predictor of Treatment Outcome

In this paper we have presented a study of the combinatorial effect of Taxol treatment and different extrinsic parameters on drug response and proliferation. Hence, the role of proliferation in the effect of certain extrinsic parameters could also be determined. Firstly, it was shown that both proliferation and drug response decreased when cells were cultured as multilayer clusters. This indicates that there was a direct relationship between the effect of the 3D culture on drug response and proliferation. Similarly, increased cell density also resulted in a decrease in both drug response and proliferation. Although the absolute differences in drug response and proliferation were different, the relative values were in the same range, i.e., the 1.4 fold increase in drug response in 2D vs. 3D correlated with a 1.3 fold increase in proliferation. This fact suggests that both three-dimensionality and cell density affect drug responses predominantly through alterations in the regulation of the cell cycle. Interestingly, previous research has shown that increased cell-cell adhesion in multicellular spheroids affects cell cycle regulation by increased levels of cyclin dependent kinase inhibitor p27 [13].

In contrast, interfering with matrix adhesion by blocking β1-integrin function was shown to increase drug response, while it induced a reduction in proliferation. This inverse relationship revealed that the effect of matrix adhesion on drug response was not proliferation-dependent but may instead be attributed to cell death signaling downstream of integrin ligation. Matrix adhesion is not only known to cause increased cell cycle progression [39] but also anti-apoptosis signaling. Indeed, adhesion-mediated increase of Akt (serine/threonine Kinase) phosphorylation has been shown to reduce the apoptosis levels induced by drug treatment [11], [53], [54].

The study of the relationship between proliferation and drug response provides intriguing insights into the signaling pathways occurring in different microenvironmental settings. These results could be of interest for the development of new treatment prediction methods. The pharmaceutical industry is shifting towards personalized treatment, as it is forecasted to greatly increase efficiency. To enable the implementation of personalized therapy, reliable biomarkers are needed [55]. Proliferation index is one phenotype-related marker that has been highlighted as a possible predictor. Many anti-cancer drugs are most effective in proliferating cells, where the contrasting example are dormant cells, and in cancer instigating cells, which may survive cytotoxic treatment [56] and form micro-metastases years later. However, it has been shown that proliferation values cannot always predict the response to chemotherapeutics [57], [58]. One explanation for this inconsistency could be the effect of microenvironmental parameters with multivariate influence on signaling pathways in cell growth and survival as highlighted by our results. Therefore, although proliferation index can be useful, the combination with another marker will probably give a better prediction of drug response [10]. E-cadherin is known to play an important role in cancer, and its down regulation is associated with increased invasiveness. In this paper we show that E-cadherin levels directly correspond to cell proliferation and drug response in breast cancer. Therefore, combining E-cadherin-levels with proliferation index should give enforced strength to the predictive value of proliferation index. On the other hand, our work shows an inverse relationship between proliferation and drug response with integrin activation. Therefore, specific integrin expressions could also constitute putative complementary markers to proliferation.

### Conclusions

In summary, this work showed that a microwell array based on a PEG hydrogel could be used to create a model of early cancer. In this model, several parameters of the tumor microenvironment, including matrix interface and cell-cell contacts, could be controlled. By the use of confocal imaging and sub-cellular resolution, drug response could be determined and correlated with the spatial location of the cell within clusters in the microarray. Therefore, it was possible to differentiate between cells in contact with predominately the ECM or with other cells.

With this model we could observe that matrix-induced drug response plays an important role in multilayered cells. In addition, it was confirmed that this effect was independent of other parameters, such as cell morphology and density. On the other hand, cell density was shown to be an additional and independent determinant of drug response. Studying different cell densities in monolayer cell clusters revealed that cell density was largely responsible for the effect multilayered cell culture had on both drug response and proliferation. A direct relationship between drug response and proliferation with cell density changes was observed, which could be correlated to increased E-cadherin levels at higher cell density. On the contrary, this was not observed for matrix dependent changes of drug response. These results indicate that both cell cycle regulation and cell death signaling are involved in determining the drug response in this model of early breast cancer.

Perhaps the most important conclusion of this study is that relatively simple models, like the one presented herein, could serve as complementary tools for pre-clinical development as physiologically relevant models that deliver clear results with high reproducibility.

## Materials and Methods

### Materials and Cells

The poly(ethylene glycol) (PEG) polymers PEG-vinyl sulfone (VS) and PEG-thiol (SH) were both from the lab of Matthias Lütolf, EPFL, Switzerland. Tissue culture treated μ-Slide 8-well dishes were purchased from Ibidi, Germany. Collagen I was purchased from Gibco, Switzerland. Laminin was obtained from Sigma, Germany and labeled with a maleimide-PEG-N-hydroxysuccinimide ester (JenKem Tech, USA) before use. The protein solutions were diluted to a working concentration of 300 µg/ml in PBS before use.

MCF-7 and MDA-MB-231 cells were purchased from the American Type Culture Collection (ATCC, Manassas, VA, USA). Dulbecco’s modified Eagle’s medium (DMEM) cell culture media, penicillin/streptomycin and FBS were all obtained from Invitrogen, Switzerland. Taxol was purchased in 1 mg aliquots from Sigma Aldrich and stored as 1 mM stock solutions in DMSO at −20°C. The mAb13 β1-integrin blocking antibody was a kind gift from Kenneth Yamada, NIH/NIDCR, Bethesda, MD. The engineered silencer nucleic acids (s-2769 and s-control) for the E-cadherin knockdown experiment were from Ambion, USA.

### Fabrication of the PEG Microwell Arrays

Microwell arrays were produced at the bottom of 8-well Ibidi dishes by micromolding as described previously [38]. In short, the microwell arrays were prepared by molding the PEG hydrogel on a poly(dimethylsiloxane) (PDMS) mold. To create protein coatings only at the bottom of the microwells, protein was printed onto the top of a microstructured PDMS mold using a wet microcontact printing process as described in [37] (Fig. S1A). Briefly, 200 µl of a protein solution (0.3 mg/ml) was placed on top of a flat polyacrylamide hydrogel. After drying, the protein-coated gel was placed in close contact with the PDMS mold to allow protein transfer. The types of matrices used (i.e., collagen I, fibronectin or laminin) were incorporated into the PEG hydrogel using two different strategies. Collagen I and laminin were especially used in this study. The large collagen molecule was applied unmodified and it incorporated into the gel by polymerization during the gel molding step. Both laminin and fibronectin was first conjugated to a PEG-malemide linker to allow sufficient attachment to the gel via covalent linkage.

The PEG hydrogel was prepared by combining PEG-vinyl sulfone (5% (w/v)) and PEG-thiol (5% (w/v)) to obtain a stoichoimetric ratio of 1∶1. Within a few minutes after mixing, the polymer solution (10–15 µl) was pipetted onto the microstructured PDMS stamp. Finally, the gel was molded between the PDMS stamp and the polystyrene surface of an Ibidi dish. Polymerization was achieved within 45–60 min at RT. Afterwards, the Ibidi dish was carefully removed and the arrays were covered with PBS and stored at 4°C until required. Before usage, the arrays were sterilized with UV light and incubated with PLL-*g*-PEG (200 µg/ml in PBS for 1 h) to render the tissue culture-treated (poly)styrene surface surrounding the gel non-adhesive for proteins and cells [59].

### Assessing the Role of Matrix Coating

The PEG microwell arrays were prepared the day before cell seeding. Multilayer clusters were formed by seeding cells into arrays containing circular microwells 90 µm in diameter and 80 µm deep coated with either collagen I or laminin. MCF-7 (or MDA-MB-231, see text for details) cells were seeded into the microwells at a seeding density of 1.5 × 10^5^ cells per Ibidi dish well. After 3–4 hrs, cells that had not entered the wells were removed from the plateau surface by two rinsing steps.

To determine the sensitivity to Taxol, cell clusters were treated with 100 nM Taxol (or 0.01% DMSO (v/v) in the controls) 24 hrs after seeding. After an additional 24 hrs incubation period, cells were fixed with ice-cold methanol (70% (v/v) in PBS for 20 min), rinsed with PBS twice and stained with propidium iodide by incubation in PI/RNAse staining buffer (undiluted for 30 min at RT; BD Biosciences, Switzerland). The drug response levels were determined as the number of fragmented cell nuclei versus total nuclei.

### Assessing the Role of β1-integrin

The clusters were formed in the microwells as described above. To optimize seeding, we centrifuged the cells (7×10^4^ cells per Ibidi dish well) at 1000 rpm into the microwells. To assess the role of β1-integrin function in the reduced drug response on collagen I, we used the well-characterized monoclonal antibody 13 (mAb13) that binds to β1-integrin and favors its inactive conformation [42]. The mAb13 antibody was added 24 hrs after cluster formation at a concentration of 50 µg/ml. Following 24 hrs culture in the presence of the antibody, cells were either assessed for proliferation or treated for an additional 24 hrs with a mixture of 50 µg/ml mAb13 and 100 nM Taxol or mAb13 alone, while control samples lacked either taxol, mAb13 or both reagents.

### Assessing the Role of Cell Density and E-cadherin

The multilayer clusters were formed in the microwells as described above. To create monolayer cell clusters on protein islands, we seeded 2 × 10^4^ cells per array with 200 µm wide, collagen I-coated microwells. After centrifugation, this density resulted in patterns with varying cell density, from very low to confluent, as confirmed by confocal microscopy. After 48 hrs culture in 2D or 3D, the cells were either assessed for proliferation or treated for an additional 24 hrs with 100 nM Taxol.

To modulate cell to cell interactions, E-cadherin expression in MCF-7 cells was knocked down by transfection with E-cadherin si-RNA. Lipofectamine-2000 (Invitrogen) was mixed with Opti-Mem (1∶150, 5 min at RT, Invitrogen) and subsequently combined with E-cadherin si-RNA (or scrambled control) and incubated for 15 min before addition to the cells. Cells were transfected by reverse transfection, in which transfection reagents and si-RNA are added to cells in suspension before seeding onto substrates. The final concentration of cells was 7×10^4^ cells per 300 µl cell culture media and 100 µl Optimem. The final concentration of Lipofectamine and si-RNA was 1∶1200 and 20 nM respectively. The samples were incubated for 24 hrs with transfection media. After an additional growth period of 24 hrs in normal culture media, the cells were assessed for proliferation or treated for 24 hrs with 100 nM Taxol.

To stain for E-cadherin, cell samples were first fixed with PBS containing 3% paraformaldehyde (w/v) for 10 min and subsequently permeabilized with Triton-X 100 (0.1% (v/v), 10 min). Before adding the primary antibodies, samples were rinsed twice with PBS and blocked with 1% BSA for 30 min. Subsequently, samples were incubated with primary anti-E-cadherin antibody (1∶100, BD Biosciences) overnight at 4°C. After rinsing, the samples were incubated with secondary Alexa-Fluor 488-conjugated anti-mouse antibody (1∶500, 1 h at RT) and counterstained with Hoechst 33342 (1∶3000, 1h RT) before a final rinse and imaging.

### Determining Proliferation by BrdU Incorporation

Proliferation was assessed using the BrdU assay as described previously [35]. Following an overnight incubation period, the media was exchanged for media containing 10 µM BrdU (Sigma Aldrich, Switzerland) and the samples were incubated at 37°C for an additional 6 hrs; subsequently all the samples were fixed with ice-cold methanol (70% (v/v) in PBS for 20 min). The samples were treated with 2 M HCl for 20 min, neutralized with 0.1 M Borax for 2 min and permeabilized with 0.1% (v/v) Triton-X for 10 min. Samples were labeled with primary mouse-anti-BrdU IgG (1∶100 (v/v); 60 min; BD Biosciences), washed three times with PBS and incubated with goat anti-mouse IgG Alexa Fluor 488 (1∶400 (v/v); 60 min; Molecular Probes). 0.5% (w/v) BSA was included in all staining buffers to avoid non-specific binding. The samples were counterstained with PI/RNAse buffer (undiluted, 30 min), to label cell nuclei, and imaged using confocal microscopy as described above. BrdU-labeled versus PI-labeled nuclei were manually counted. All stages following fixation were performed at room temperature.

### Imaging of Single Cells in Multilayer Cell Clusters

Confocal microscopy was employed to allow imaging of the clustered cells with single cell resolution. Two confocal microscopes containing water immersion objectives were used: a Leica SP2-AOBS CLSM (Leica, Germany) with a ×20 objective, NA = 0.7 and an Olympus FV microscope (Olympus, Japan) with a ×40 objective, NA = 0.9. To acquire information from different positions within the cell clusters with restrained experimental variability, three individual z-sections were acquired per cell cluster in all experiments; z1, z2 and z3, where z1 represented the cells in direct contact with the matrix (i.e., bottom location). The z-image planes were separated by ∼15 µm to avoid overlapping assessments (Fig. 1A).

### Statistic Analysis

All experiments were performed in duplicates and repeated three times. Quantitative data was plotted as the mean ± standard error of the mean (SEM). Statistical analysis was performed using Student’s unpaired two-way t-tests. Differences were considered to be statistically significant when p<0.05.

## Supporting Information

Figure S1 **Fabrication of the protein-coated microwell arrays.** A) A PDMS master is molded on a Si-wafer with SU8 microstructures (i). In the next step, this master is coated with matrix coating at the top of the pillars (ii). A wet microcontact printing method is used to obtain a reliable protein transfer. The protein of choice is dried on top of a polyacrylamide gel, and then this gel is placed upside down on the PDMS master. Finally a small volume of PEG-gel precursor is placed on the PDMS master and molded into a thin film between the PDMS master and the TCP substrate (iii). Upon removal from the PDMS master, the gel will stick to the TCP surface and hence make up a suitable cell culture substrate (iv). B) The protein coating is visualized by indirect fluorescence for Fn, Lam and Col-I. Lam and Col-I are stained with respective primary antibodies and visualized by secondary antibody staining using an Ab-Alexa Fluor 488 conjugate while the Fn-coating was visualized by immobilizing Fn directly conjugated to Alexa Fluor 488.
(TIF)

Figure S2 **Cell density and proliferation in the microwells for MDA-MB-231 and MCF-7.** We found significant differences in growth behavior and in the packing density when the two examined cell lines MCF-7 and MDA-MB-231 were grown in 90 µm wide collagen I-coated microwells (A). The images show cell nuclei stained with propidium iodide (red) and antibody for BrdU incorporation indicating DNA synthesis (green) (MUCL  =  multilayer cell cluster). After 72 h culture of these cancer cells in microwells, proliferation was reduced in comparison to on collagen I-coated TCPS. The effect was significantly greater in the MDA-MB-231 cells (B). At this point the cells in the MCF-7 clusters were significantly denser than the cells in the MDA-MB-231 clusters (C). (* = p<0.05).
(TIF)

Figure S3 **Microscopy-based read-out of experiments in the cell cluster microarray.** MCF-7 cells seeded into the microwell array form clusters with a narrow size distribution. Because clusters are aligned in the same z-plane, the imaging can be performed in an automated manner. The width of the MCF-7 multilayered cell clusters was found to be 80–90 µm and 45–50 µm for wells with a diameter of 90 and 50 µm respectively. The height of the clusters could be tuned by seeding conditions and culture time. To measure cluster heights, we stained the cells’ actin cytoskeleton using fluorescently pre-labeled phalloidin and analyzed the average cluster heights by means of confocal microscopy. Results suggested an average height of 56±3 µm at 48 hr after seeding 1.5×10^5^ cells onto arrays of microwells with a diameter of 90 µm. The thin hydrogel allowed us to use confocal imaging, collecting information at three different image planes; z1, z2 and z3. This enabled evaluation of cell behaviour at the single cell level. The lower right image shows nuclear fragmentation within a cluster, which was used to read out apoptosis after treatment with Taxol. Scale bar is 50 µm.
(TIF)

Figure S4 **The effect of blocking β1-integrin at different positions within the multilayer clusters.** Blocking integrin β1 has a strong effect on drug response, but this effect was only significant in the two image planes closest to the collagen I coating (A). The treatment also affected proliferation. The z-plot revealed that the effect was greatest in image planes z1 and z2, while there was no significant difference in proliferation at the z3 location (B). Furthermore, it could be confirmed that these effects were independent of cell density, as no significant differences in cell density between image planes were observed following β1-integrin inhibition (C). (* and *** represent p<0.05 and p<0.001 respectively, n.s.  =  not significant).
(TIF)
