# Supplementary figures and images for: Controlled Breast Cancer Microarrays for the Deconvolution of Cellular Multilayering and Density Effects upon Drug Responses
Source: PLoS One. 2012 Jun 29;7(6):e40141. doi: 10.1371/journal.pone.0040141 (PMC3387021; doi:10.1371/journal.pone.0040141)

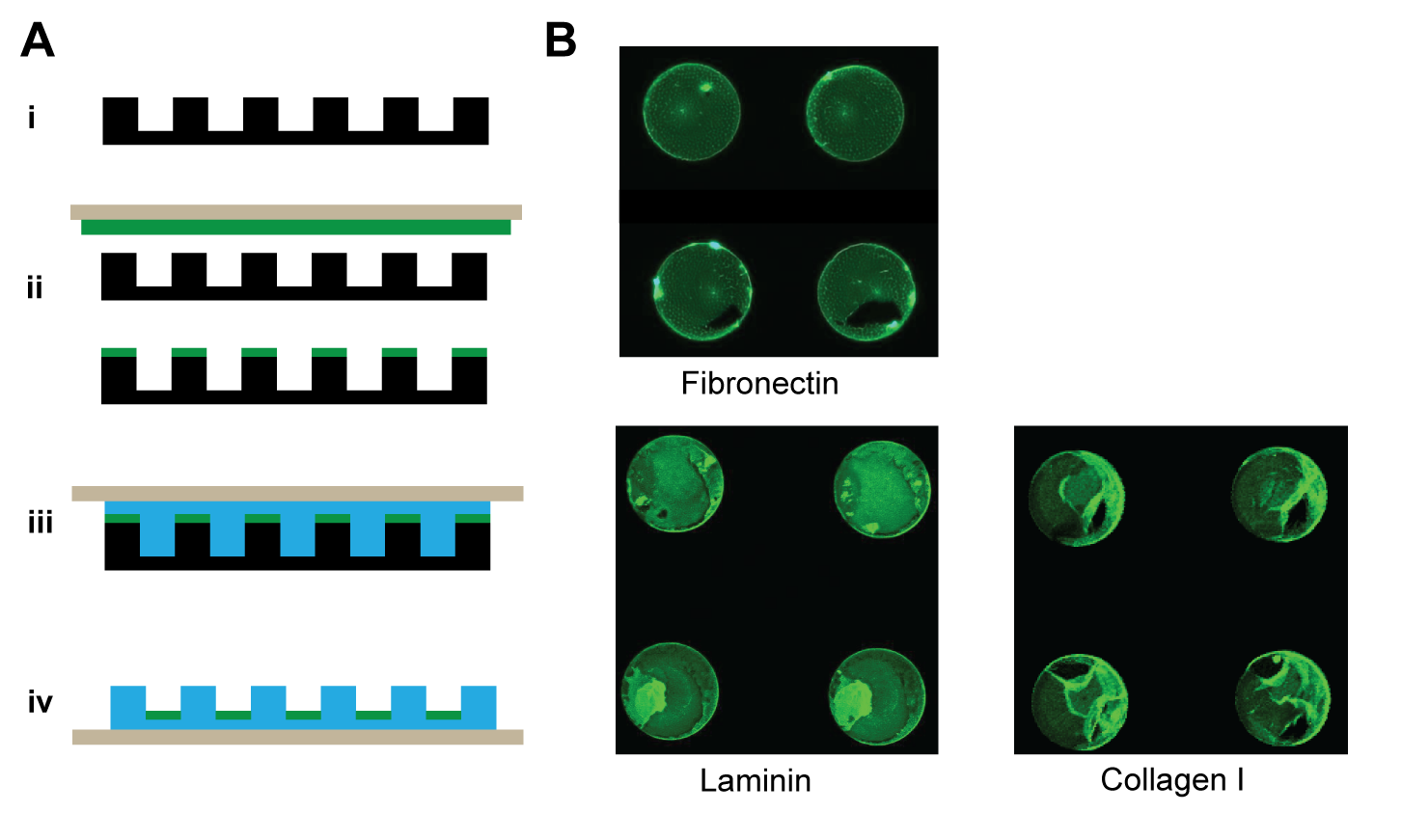

Supplement: Figure S1 — Fabrication of the protein-coated microwell arrays. A) A PDMS master is molded on a Si-wafer with SU8 microstructures (i). In the next step, this master is coated with matrix coating at the top of the pillars (ii). A wet microcontact printing method is used to obtain a reliable protein transfer. The protein of choice is dried on top of a polyacrylamide gel, and then this gel is placed upside down on the PDMS master. Finally a small volume of PEG-gel precursor is placed on the PDMS master and molded into a thin film between the PDMS master and the TCP substrate (iii). Upon removal from the PDMS master, the gel will stick to the TCP surface and hence make up a suitable cell culture substrate (iv). B) The protein coating is visualized by indirect fluorescence for Fn, Lam and Col-I. Lam and Col-I are stained with respective primary antibodies and visualized by secondary antibody staining using an Ab-Alexa Fluor 488 conjugate while the Fn-coating was visualized by immobilizing Fn directly conjugated to Alexa Fluor 488. (TIF) [file pone.0040141.s001.tif]

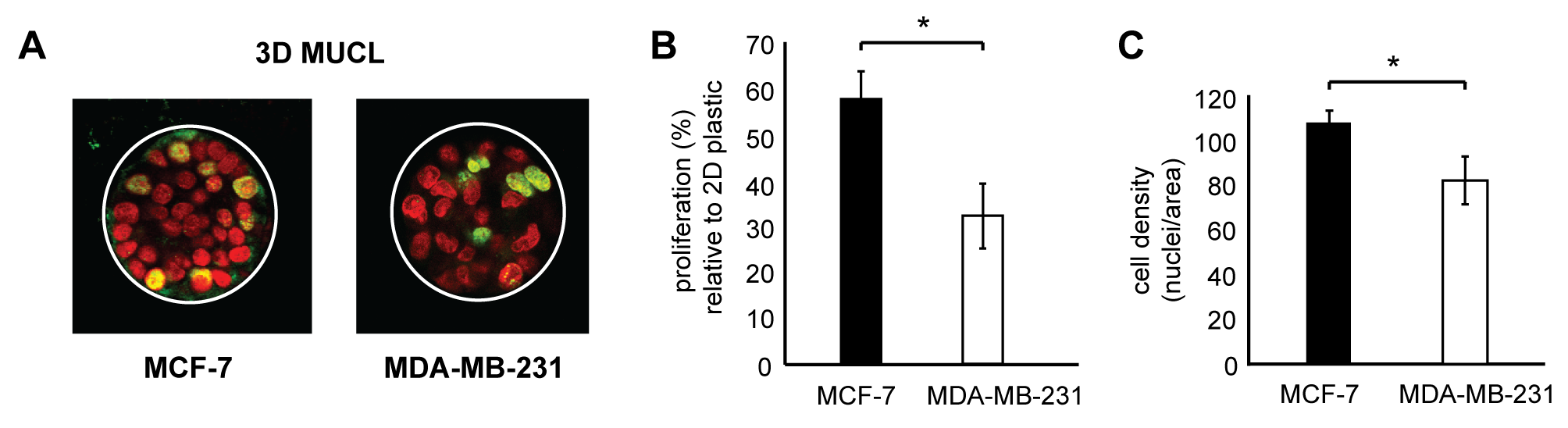

Supplement: Figure S2 — Cell density and proliferation in the microwells for MDA-MB-231 and MCF-7. We found significant differences in growth behavior and in the packing density when the two examined cell lines MCF-7 and MDA-MB-231 were grown in 90 µm wide collagen I-coated microwells (A). The images show cell nuclei stained with propidium iodide (red) and antibody for BrdU incorporation indicating DNA synthesis (green) (MUCL = multilayer cell cluster). After 72 h culture of these cancer cells in microwells, proliferation was reduced in comparison to on collagen I-coated TCPS. The effect was significantly greater in the MDA-MB-231 cells (B). At this point the cells in the MCF-7 clusters were significantly denser than the cells in the MDA-MB-231 clusters (C). (* = p<0.05). (TIF) [file pone.0040141.s002.tif]

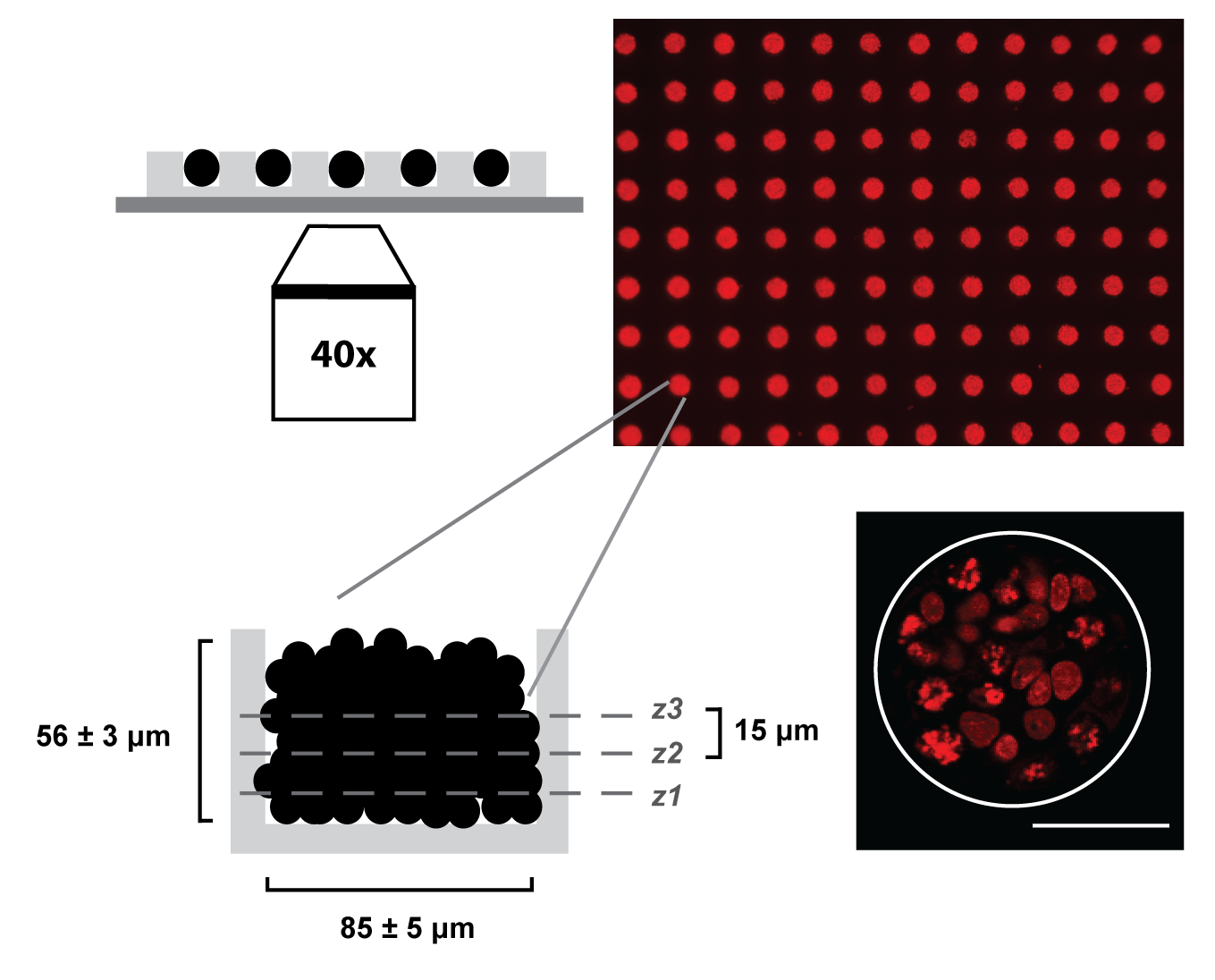

Supplement: Figure S3 — Microscopy-based read-out of experiments in the cell cluster microarray. MCF-7 cells seeded into the microwell array form clusters with a narrow size distribution. Because clusters are aligned in the same z-plane, the imaging can be performed in an automated manner. The width of the MCF-7 multilayered cell clusters was found to be 80–90 µm and 45–50 µm for wells with a diameter of 90 and 50 µm respectively. The height of the clusters could be tuned by seeding conditions and culture time. To measure cluster heights, we stained the cells’ actin cytoskeleton using fluorescently pre-labeled phalloidin and analyzed the average cluster heights by means of confocal microscopy. Results suggested an average height of 56±3 µm at 48 hr after seeding 1.5×10^5^ cells onto arrays of microwells with a diameter of 90 µm. The thin hydrogel allowed us to use confocal imaging, collecting information at three different image planes; z1, z2 and z3. This enabled evaluation of cell behaviour at the single cell level. The lower right image shows nuclear fragmentation within a cluster, which was used to read out apoptosis after treatment with Taxol. Scale bar is 50 µm. (TIF) [file pone.0040141.s003.tif]

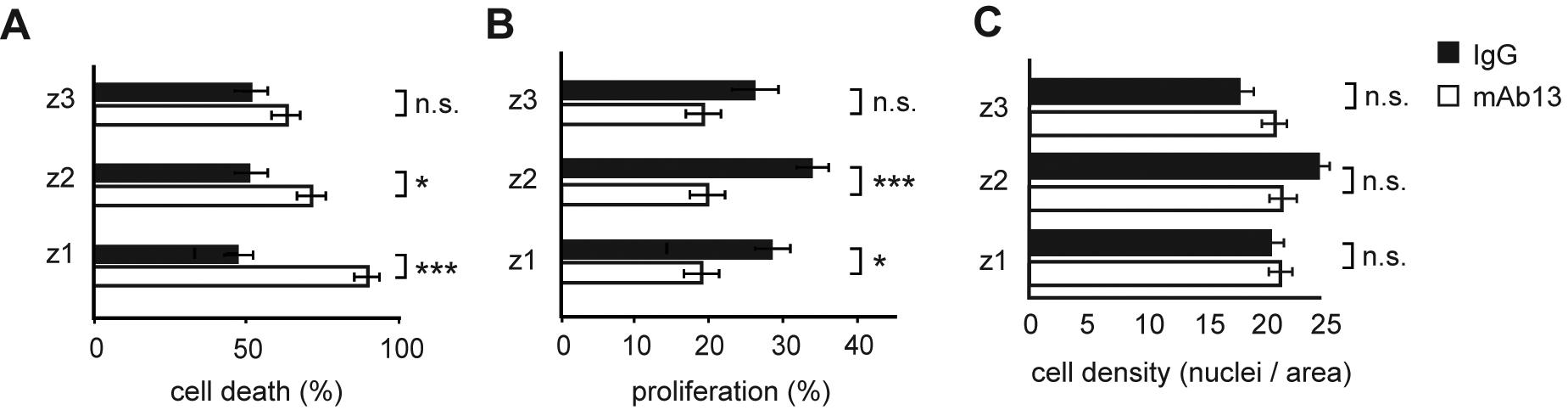

Supplement: Figure S4 — The effect of blocking β1-integrin at different positions within the multilayer clusters. Blocking integrin β1 has a strong effect on drug response, but this effect was only significant in the two image planes closest to the collagen I coating (A). The treatment also affected proliferation. The z-plot revealed that the effect was greatest in image planes z1 and z2, while there was no significant difference in proliferation at the z3 location (B). Furthermore, it could be confirmed that these effects were independent of cell density, as no significant differences in cell density between image planes were observed following β1-integrin inhibition (C). (* and *** represent p<0.05 and p<0.001 respectively, n.s. = not significant). (TIF) [file pone.0040141.s004.tif]
